# Structural characterization of alkaline hydrogen peroxide pretreated grasses exhibiting diverse lignin phenotypes

**DOI:** 10.1186/1754-6834-5-38

**Published:** 2012-06-06

**Authors:** Muyang Li, Cliff Foster, Shantanu Kelkar, Yunqiao Pu, Daniel Holmes, Arthur Ragauskas, Christopher M Saffron, David B Hodge

**Affiliations:** 1 Department of Biosystems and Agricultural Engineering, Michigan State University, Michigan, USA; 2 DOE Great Lakes Bioenergy Research Center, Michigan State University, Michigan, USA; 3 Department of Chemical Engineering and Materials Science, Michigan State University, Michigan, USA; 4 DOE BioEnergy Science Center, Georgia Institute of Technology, Georgia, USA; 5 Department of Chemistry, Michigan State University, Michigan, USA; 6 Department of Chemistry and Biochemistry, Georgia Institute of Technology, Georgia, USA; 7 Institute of Paper Science and Technology, Georgia Institute of Technology, Georgia, USA; 8 Department of Forestry, Michigan State University, Michigan, USA; 9 Department of Chemical Engineering and Materials Science, Michigan State University, Michigan, USA

**Keywords:** Alkaline hydrogen peroxide pretreatment, Cellulosic biofuels, Lignin, Plant cell wall analysis, Analytical pyrolysis

## Abstract

**Background:**

For cellulosic biofuels processes, suitable characterization of the lignin remaining within the cell wall and correlation of quantified properties of lignin to cell wall polysaccharide enzymatic deconstruction is underrepresented in the literature. This is particularly true for grasses which represent a number of promising bioenergy feedstocks where quantification of grass lignins is particularly problematic due to the high fraction of *p-*hydroxycinnamates. The main focus of this work is to use grasses with a diverse range of lignin properties, and applying multiple lignin characterization platforms, attempt to correlate the differences in these lignin properties to the susceptibility to alkaline hydrogen peroxide (AHP) pretreatment and subsequent enzymatic deconstruction.

**Results:**

We were able to determine that the enzymatic hydrolysis of cellulose to to glucose (*i.e.* digestibility) of four grasses with relatively diverse lignin phenotypes could be correlated to total lignin content and the content of *p*-hydroxycinnamates, while S/G ratios did not appear to contribute to the enzymatic digestibility or delignification. The lignins of the brown midrib corn stovers tested were significantly more condensed than a typical commercial corn stover and a significant finding was that pretreatment with alkaline hydrogen peroxide increases the fraction of lignins involved in condensed linkages from 88–95% to ~99% for all the corn stovers tested, which is much more than has been reported in the literature for other pretreatments. This indicates significant scission of β-O-4 bonds by pretreatment and/or induction of lignin condensation reactions. The S/G ratios in grasses determined by analytical pyrolysis are significantly lower than values obtained using either thioacidolysis or 2DHSQC NMR due to presumed interference by ferulates.

**Conclusions:**

It was found that grass cell wall polysaccharide hydrolysis by cellulolytic enzymes for grasses exhibiting a diversity of lignin structures and compositions could be linked to quantifiable changes in the composition of the cell wall and properties of the lignin including apparent content of the *p*-hydroxycinnamates while the limitations of S/G estimation in grasses is highlighted.

## Background

Biofuels and green chemicals from plant lignocellulose offer the promise of decreased petroleum consumption with the caveat that technological and economic barriers remain that impede large-scale industrial implementation. The polysaccharide portion of plant cell walls (cellulose and hemicellulose) is ideally suited to conversion *via* biochemical transformations due to the central placement of carbohydrates in cellular metabolism. A diverse set of enzymes and metabolic pathways currently exist that are capable of converting carbohydrates to a wide range of useful metabolites and recent progress in metabolic and protein engineering is expanding this range. The challenge to realizing the potential of plant cell wall polysaccharides is primarily due to the set of plant properties collectively known as “biomass recalcitrance” [1] that limit the availability of polysaccharides for biological conversion by enzymatic or catabolic routes. This recalcitrance is primarily derived from the heterogeneous supramolecular organization of the plant cell wall matrix or higher order structures in the plants and necessitates a chemical or physical pretreatment step prior to biological conversion [2]. These higher order structures include considerations such as overall plant anatomy, cell wall thickness, covalent and non-covalent interactions between macromolecules (cellulose, hemicellulose, and lignin) as well as distribution of these macromolecules within the cell wall matrix.

Polysaccharides within secondary cell walls are embedded within a matrix of lignin that limits their accessibility. Lignin’s physiological role in the plant cell wall and the reason for its contribution to recalcitrance is to protect vulnerable carbohydrates from attack by pathogens, provide structural stability to the cell wall, and present a hydrophobic barrier to water penetration through cell types that serve the purpose of fluid transport. While lignin’s role in cell wall recalcitrance is universally accepted, the precise set of factors that contribute to this recalcitrance are not universally acknowledged. Factors specific to lignin’s role in recalcitrance have been proposed to include the total lignin abundance [3-5], lignin location within the cell wall [6], and the properties of lignin such as hydrophobicity [7], as well as indirect impacts such as lignin’s ability to bind enzymes [8].

Lignin is a polymer composed primarily from three “canonical” *p*-hydroxycinnamyl alcohols (monolignols) including coniferyl, sinapyl, and to a lesser extent *p-*coumaryl alcohols which form the guaiacyl (G), sinapyl (S), and *p*-hydroxyphenyl (H) units in lignin [9]. Other “non-canonical” aromatic monomers taken from other points of the monolignol biosynthesis grid are known to be incorporated into the lignin polymeric framework as well [10,11]. The linkages between monolignols can be through ethers such as the β-O-4 (β-aryl ether) and 5-O-4 (biphenyl ether) linkages, C-C (“condensed”) bonds such as 5–5 (biphenyl) linkages, or a combination of C-C and ether linkages as in β–5 + α-O-4 (phenylcoumaran) and β–β + γ-O-α + α-O-γ (resinol) among others [12], as well as ester linkages involving, for example, *p*-hydroxycinnamates [13]. The total content and relative abundance of monolignols, their linkages, and degree of crosslinking with polysaccharides varies by plant taxa, plant developmental stage, and plant tissue [14-16]. Other features of lignin that differ between plant source include potentially the degree of polymerization (although this is not clear) and the number of free phenolic groups. Free phenolics provide initiation sites for alkaline and oxidative delignification and contribute to lignin’s alkali solubility [17,18] due to deprotonation of phenolic hydroxyls at a lower pH than aliphatic hydroxyls.

A significant number of promising bioenergy feedstocks are commelinid monocots, specifically the *Poaceae* or grasses, and include agricultural wastes [19] such as corn stover, wheat straw, rice straw, and sugar cane bagasse and dedicated perennial energy crops such as switchgrass and *Miscanthus* spp. among others. Lignin composition and cell wall structural organization in grasses is significantly different from herbaceous and woody dicots (forbs and hardwoods, respectively) or gymnosperm lignins. One distinguishing feature of the monocot lignins is the considerable incorporation of the *p*-hydroxycinnamic acids including ferulic and *p*-coumaric acids [13,20,21]. Ferulate monomers and dimers are known to be ester-linked to glucuronoarabinoxylan [22] and are proposed to be involved in ether and C-C linkages to the lignin polymer that act as crosslinks between hemicellulose and lignin polymer chains [11,13,23]. Monomers of *p-*coumaric acid are proposed to be esterified to the lignin polymer at the γ-carbon of the side chain region of β-O-4 linked syringyl moieties [24] and to a lesser degree esterified to glucuronoarabinoxylan. Grass lignins are significantly more condensed (*i.e.* contain more C-C linkages between monolignols) and have higher phenolic hydroxyl contents than the lignins of dicots [21,25] and an important implication of this is that more than 50% of grass lignins can be solubilized by treatment with alkali [26] due to the destruction of alkali-labile ester linkages along with the high free phenolic content improving lignin solubility in alkali [17].

Plants have typically neither been under selective evolutionary constraint nor bred to yield phenotypes that would yield high polysaccharide conversion for a bioenergy process, although the identification and propagation of forage crops with the phenotype for high digestibility in ruminants [27,28] represents an important starting point. Examples include forage improvement studies on corn stover [28] and the identification of the brown midrib mutations in grasses including maize, millet, and sorghum [29] which have been known as having the phenotype for improved ruminant digestibility in corn for more than 50 years [30-32]. The brown midrib lines of maize are known to contain less lignin as well as altered monolignol ratios, and altered inter-monolignol linkages which the present work aims to exploit in comparing differences in the lignin contents and structures.

Alkaline hydrogen peroxide (AHP) pretreatment has been studied as a chemical pretreatment [33-36] and as a delignifying post-treatment [37,38] and is based on treatment of biomass with hydrogen peroxide at alkaline pH (optimally at pH 11.5) at ambient or near-ambient temperatures and pressures. Due to the distinctive properties of their lignins and structural organization of their cell walls as described above, alkaline pretreatments are particularly effective for grasses, and it is known that AHP is less effective on forbs [36] and woody dicots (unpublished observations). We hypothesize that the cellulose enzymatic digestibility improvement resulting from AHP pretreatment may be attributable to the destruction of ferulate crosslinks as well as mild oxidation and solubilization of lignin. These outcomes have the net effect of improving the overall hydrophilicity of the cell wall matrix which can allow for water and hydrolytic enzyme penetration.

In this work, AHP pretreatment is used to generate a set of biomass samples exhibiting a diverse range of lignin contents and abundance of the *p*-hydroxycinnamates with the goal of better characterizing the relationship between a delignifying pretreatment, the composition and properties of the cell wall, and the digestibility by cellulolytic enzymes in grasses displaying a diverse natural range of lignin phenotypes. These lignin phenotypes include differences in total lignin content, differences in the relative abundance of monolignols incorporated into the cell wall with the consequence of altered linkages between monolignols, and differences in the ferulate content resulting in differences in the extent of cross-linking between cell wall polymers. Specifically, these biomass types consist of a switchgrass cultivar, a commercial hybrid corn stover, and two inbred brown midrib lines of maize (bm1 and bm3). The relationship between S/G ratios as determined by Py-GC/MS, thioacidolysis-GC/MS, and HSQC NMR are compared in parallel for the first time for grasses, while HSQC NMR is applied to characterize structural changes associated with the whole cell wall, with the overall intention of identifying and linking properties associated with grass lignins to improved cellulolytic enzyme digestibility.

## Results

### Relationship between pretreatment, composition, and glucan enzymatic digestibility

**Figure 1:**
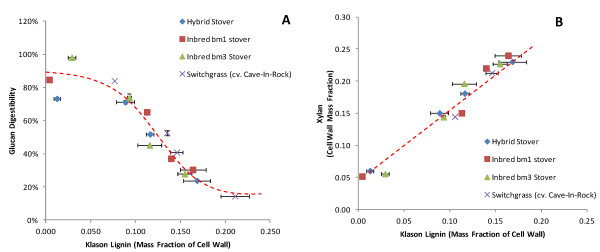
Correlating Klason lignin content of the cell wall to glucan digestibility for all pretreatment conditions and biomass types.

**Figure 2:**
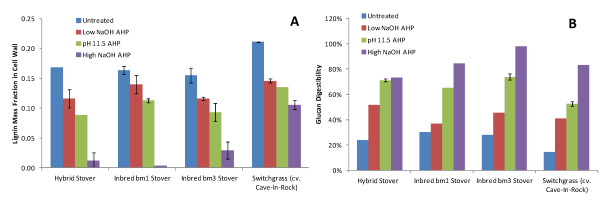
Data for (A) Klason lignin and (B) glucan digestibility as a function of pretreatment condition.

Figures 1 and 2 depict the changes in glucan enzymatic digestibility and composition as a function of pretreatment condition and degree of delignification. Figure 1 shows a clear negative relationship between lignin content and glucan digestibility that can be approximately described by a sigmoidal function. The rapid change in the glucan digestibility slope between a lignin content of 10% and 15% may indicate other changes in the cell wall rather than just total lignin content. Alternatively, this may indicate a “threshold” value for lignin removal that is necessary for either enzyme/water penetration into the cell wall or improves access into the cell wall by removing lignin that occludes access to the bulk of the cellulose.

Data plotted in Figure 2 represents the same data as in Figure 1 re-plotted to distinguish pretreatment condition. This shows an obvious ranking of the biomass types by both lignin removal and glucan digestibility, with the general trend that the corn stovers and, in particular, the lower lignin stovers show both improved glucan digestibility and lignin removal relative to the switchgrass, which can be considered more recalcitrant to both pretreatment and hydrolysis. Additionally, these data show that while the values differ, the responses of the four biomass types show similar trends.

### Lignin characterization by py-GC/MS

Analytical pyrolysis is used to characterize the pools of pyrolytically labile aromatics to identify the changes to lignin composition associated with pretreatment, although, as in thioacidolysis, it is thought that primarily ether (and ester) bonds are broken which results in lower monomer yields from highly condensed lignins such as grasses. Challenges for using analytical pyrolysis as a tool for characterizing plant cell wall chemical structures include the difficulty in making quantitative comparisons between samples and in making an unambiguous mapping of aromatic pyrolysis products to monomeric subunits within cell wall polymers since pyrolytic products may have more than one chemical origin. It is well-established that the *p*-hydroxycinnamic acids yield clearly identifiable markers from pyrolysis: 4-vinylphenol and 4-vinylguaiacol derived from *p-*coumaric acid (*p*CA) and ferulic acid (FA) residues, respectively [27,39,40]. However 4-vinylguaiacol may also be a marker for guaiacyl lignins involved in β-O-4 bonds [41,42] or for a terminal aliphatic coniferyl alcohol. Modification of the aliphatic region during pyrolysis or during oxidative coupling in lignification may also render ferulates and guaiacyl lignins indistinguishable (*e.g.* as guaiacol and creosol), further confounding the distinction between these two pools of monomers. While 4-vinylphenol may serve as a suitable marker for *p-*coumaroyl esters, the discrimination between pyrolytic phenol deriving from *p*-hydroxyphenyl (H) monolignols, other demethoxylated aromatics, and *p*-coumaroyl esters cannot be guaranteed. Additionally, the amino acid tyrosine may contribute to phenol (our proposed marker for H-lignins) and 4-vinylphenol [39,40]. Aromatic compounds identified in the pyrograms are presented in Table 1 with peak assignments based on comparison of mass spectra with the reference library as well as comparison with the literature [39]. Each compound is identified as likely to originate from a *p*-hydroxyphenyl (H) lignin monomer, guaiacyl (G) lignin monomer, syringyl (S) lignin monomer, *p*-coumarate (*p*CA), or ferulate (FA).

**Table 1:** Biomass-Derived Aromatic Monomers Quantified by Py-GC/MS

	Retention Time (min)	Major Mass Fragment (m/z)	Primary Origin	Secondary Origin
Phenol	16.44	94	H	*p*CA
Guaiacol	16.96	124	G	FA
Creosol	19.02	138	G	FA
p-ethylguaiacol	20.61	152	G	FA
4-vinylphenol	21.45	120	*p*CA	H
4-vinylguaiacol	21.67	150	FA	G
Syringol	22.57	154	S	-
(E)-isoeugenol	23.98	164	G	FA
Vanillin	24.49	152	G	FA
Acetoveratrone	26.27	180	G	FA
Methoxyeugenol	28.24	194	S	-

**Figure 3:**
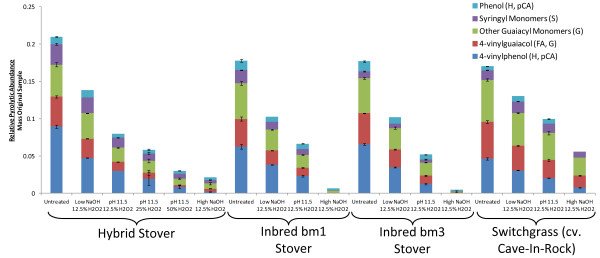
**Pools of aromatic pyrolysis products as a function of biomass source and pretreatment.** The data were normalized to the mass of the original sample based on the quantified mass loss during pretreatment and provide an approximation of the relative abundance of the five pools of aromatics volatilized by pyrolysis.

Rather than quantitating as absolute yields, component peaks within pyrograms can be interpreted quantitatively for estimating ratios between pools of components (*e.g.* S/G ratios) using, either peak area or fractional peak area [40,43,44] as is done in this work or peak area divided by its corresponding molar mass to yield an approximate relative “molar abundance” [41,42,45]. Because in this work, we are quantifying mass loss from pretreatment it is possible to calculate a relative abundance based on the fractional peak area per original mass of biomass to give an indication of the yield of pyrolytic products from the original biomass. This is done in Figure 3 which shows the relative abundance of the 5 pools of pyrolytically liberated aromatics as a function of biomass type and pretreatment and normalized for mass loss during pretreatment. The obvious trend is that all 5 pools decrease with increasing pretreatment “severity”.

Another important finding from the data in Figure 3 is that 50–70% of the pyrolytically-derived aromatics initially present in the grasses consist of 4-vinylguaiacol and 4-vinylphenol, while the *p-*hydroxycinnamic acids may account for approximately 10–25% of grass lignins [25,46]. This implies a higher fraction of these *p*-hydroxycinnamic acids are involved in pyrolytically-labile ester and/or ether bonds than the bulk lignin which is consistent with the literature [29,42,47-49]. While not representing only FA in the cell wall, the estimated pyrolytic FA may provide a good proxy of its content in addition to standard guaiacyl monolignols.

**Figure 4:**
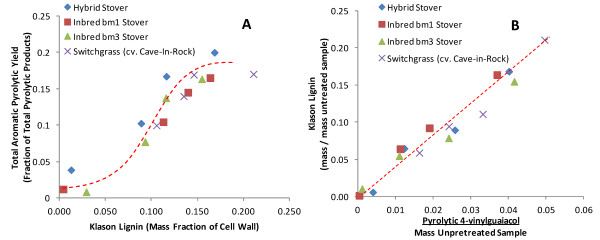
**Correlating pyrolytic yields of aromatics to Klason lignin.** A comparison of all biomass types for all pretreatment conditions shows that the relationship between **(A)** aromatic pyrolytic yield *versus* Klason lignin and **(B)** the pyrolitic 4-vinylguaiacol as a proxy for cell wall ferulates *versus* Klason lignin with both normalized to the mass of the untreated sample to indicate the amount of material removed from the cell wall.

Figure 4 gives additional insight into the changes taking place in the lignin that can be identified by analytical pyrolysis. Figure 4A shows the total aromatics liberated by pyrolysis exhibit an approximately sigmoidal relationship to the total Klason lignin content of the cell wall, which may be interpreted as representing the preferential targeting of *p-*hydroxycinnamates during AHP pretreatment such that their total apparent abundance decreases more rapidly than the bulk lignin. Previous work with analytical pyrolysis of softwoods (which do not contain ester-linked aromatics) have found linear relationships between pyrolytic aromatic fractional yield and Klason lignin [50]. Figure 4B shows a linear relationship between liberated 4-vinylguaiacol per original biomass content and Klason lignin content normalized to the original mass of biomass so that the data represent the relative amount of the original material remaining in the cell wall. This clear correlation (which is not so clear for any of the other individual pyrolytic monomer fractions) indicates, together with the data in Figure 1, that the glucan conversion is a function of lignin content and that the ferulate content is a good predictor of digestibility and lignin release.

**Figure 5:**
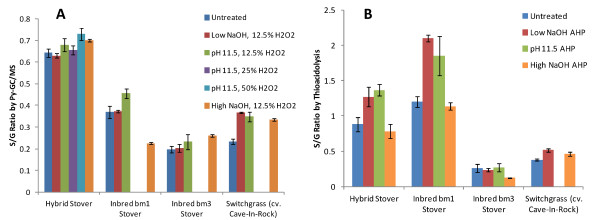
Estimated change in S/G ratio as a function of pretreatment condition using (A) analytical pyrolysis and (B) thioacidolysis.

Making additional use of the analytical pyrolysis data, Figure 5A shows estimates of S/G ratios based on the ratios of the peak areas of syringyl to guaiacyl-derived pyrolysis compounds with the exclusion of 4-vinylguaiacol. The notable trend from this figure is the minimal differences between the estimated S/G ratios as a function of pretreatment condition. Another result of Figure 5A are the clear differences in the S/G ratios between biomass types with the bm3 corn stover showing the lowest values of S/G, which is one of the known phenotypes of this line [31].

### Lignin characterization by thioacidolysis-GC/MS

Thioacidolysis is based on the cleavage of ether inter-unit linkages in lignin by boron trifluoride in ethanethiol-dioxane, followed by derivatization of the released lignin fragments and identification and quantification by GC/MS [51]. The thioacidolysis yield is dependent on the β-O-4 content of the lignin and since the β-O-4 content is dependent on S content (among others), grass lignins are known to have low monomer yields from thioacidolysis [52] relative to lignins from dicots, that typically have higher syringyl contents. Guaiacyl monolignols can be involved in much greater diversity of condensed C-C bonds at the C_5_ site on the aromatic ring and an increase in the G lignin content increases the potential for condensed bonds between monolignols, branching, and free phenolic hydroxyl groups. Additionally, the C-C linkages between monolignols are considerably more resistant to cleavage during alkaline chemical pulping [16] as well as during analytical methods such as thioacidolysis and pyrolysis [52].

Non-quantitative thioacidolysis was performed on all of the samples to generate the S/G ratios with the result plotted in Figure 5B. This shows that most of the samples have apparently higher S/G by thioacidolysis than by pyrolysis, especially for bm1 stover. One likely explanation for this discrepancy is that the ferulates which are liberated disproportionally to their abundance will contribute to guaiacyl-lignin products in pyrolysis that will artificially inflate the apparent guaiacyl-lignin content. Values obtained in the literature for typical S/G ratios of midrib and non-midrib corn stovers by thioacidolysis are slightly higher than our values [31]. Another trend from Figure 5B is that the S/G ratios increase with increasing pretreatment. It is known that *p*-coumarates are esterified primarily to the γ-position of syringyl moieties and that this acylation has been shown to reduce the thioacidolysis yield of β-O-4 linked syringyl moieties by 40% [53]. This could be a second interpretation for the trend of increasing S/G ratio as quantified by thioacidolysis, since removal of acylated *p*-coumarate during pretreatment will increase the syringyl yield during thioacidolysis. A third possible reason for the apparent enrichment in syringyl lignins by thioacidolysis for the hybrid stover and bm1 is if pretreatment is inducing condensation reactions, then only guaiacyl (and *p*-hydroxyphenyl) lignins would be able to participate in these and the corresponding thioacidolysis yield of guaiacyl and *p*-hydroxyphenyl lignins would decrease. This apparent increase in S/G ratio has been identified previously for the alkaline hydrogen peroxide bleaching of Kraft eucalypytus lignin [54].

**Figure 6:**
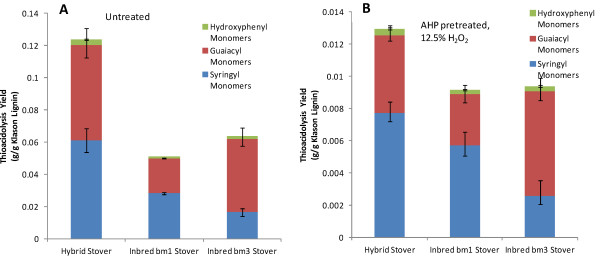
**Quantitative thioacidolysis yield for three of the three corn stovers either (A) untreated or (B) AHP pretreated at pH 11.5 12.5% H**_**2**_**O**_**2**_**loading.**

Quantitative thioacidolysis yields of the three “canonical” monolignols for the three corn stovers for either no pretreatment or AHP pretreated at pH 11.5 and 12.5% w/w H_2_O_2_ loading on the biomass are plotted in Figure 6, which shows several significant results. The first is that the overall thioacidolysis yield on lignin for the untreated hybrid corn stover is approximately 12% by mass, indicating that 88% of the lignin monomers are involved in condensed C-C coupling between monolignols while the two untreated brown midrib stovers have thioacidolysis mass yields in the range of 5–6% (Figure 6A). Explanations for this lower thioacidolysis monomer yield may be that the brown midrib stovers are either considerably more condensed or that non-canonical monolignols constitute a non-trivial fraction of the lignin, both of which would decrease the apparent yield by thioacidolysis and are supported by the literature. For example, it has been reported that bm1 and bm3 lignins are more condensed [24,31,32,55,56] than non-midrib lines. Thioacidolysis yields for corn stovers, including brown midrib lines, have been reported in a similar range to our values [24,31,49], while thioacidolysis monomer yields from hybrid poplars with varying S/G ratios in the range of 2 to 11.5 have been reported as 3–4 times higher [51] due to the higher yields from less condensed lignins

Another significant result from Figure 6B is that the AHP-pretreated corn stovers have much lower thioacidolysis monomer yields relative to the untreated corn stovers (*i.e.* 1.0–1.2% *vs.* 5–12%) indicating that the lignin remaining in the cell wall is even more condensed after pretreatment. Three scenarios that may explain this are that AHP pretreatment may be preferentially solubilizing the less condensed lignins, cleaving β-O-4 bonds, or inducing condensation reactions comparable to, for example, oxidative laccase or peroxidase-mediated lignin polymerization [57,58]. To determine whether lignin condensation was an important outcome of AHP pretreatment, a material balance on condensed lignin was performed using the data for mass loss during pretreatment, the Klason lignin content, and the thioacidolysis yield to estimate whether the total condensed lignin mass after pretreatment is greater than before pretreatment. The material balance did not show that the overall condensed lignin mass was increased so that pretreatment-induced condensation is presumably minimal.

It is known that in addition to coniferyl alcohol, bm1 lines incorporate coniferyl aldehyde into their lignins due to a presumed deficiency cinnamyl alcohol dehydrogenase (CAD), while caffeic acid O-methyltransferase (COMT) deficiency in bm3 lines results in benzodioxane structures in lignin deriving from incorporation 5-hydroxyguaiacyl moieties [59] in addition to a significantly reduced syringyl lignin content. These examples of alternate monolignol incorporation are known to yield distinct thioacidolysis products [55,60] other than what are quantified in the present work and could contribute to both lower S/G ratios and the lower total thioacidolysis yields. We were able to identify peaks in only the untreated bm1 stover thioacidolysis chromatograms (data not shown) exhibiting an m/z of 354 and 384 which are proposed to be indene markers representing incorporation of coniferaldehyde and sinapaldehyde, respectively, into the growing lignin polymer due to CAD defficiency [60]. The coniferaldehyde response was approximately 4.3% of the guaiacyl signal while the sinapaldehyde response was approximately 1.5% of the syringyl signal. These markers are only present in the untreated bm1stover which indicates these monomers are removed during AHP pretreatment.

### Lignin characterization by HSQC NMR

**Figure 7:**
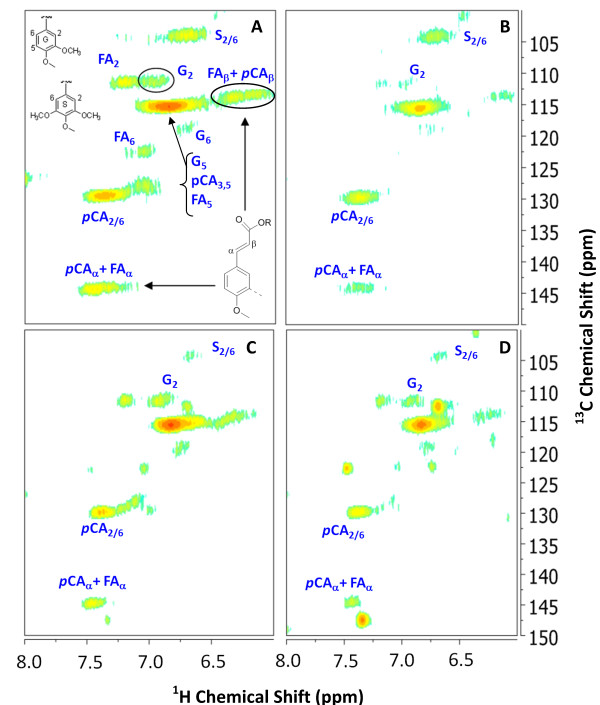
**Aromatic region of**^**13**^ **C-**^**1**^ **H HSQC spectra of (A) untreated and (B) AHP pretreated hybrid corn stover and (C) untreated and (D) AHP pretreated inbred bm3 corn stover in perdeuterated pyridinium chloride/DMSO-d**_**6**_**.**

Solution-state HSQC NMR has recently been applied as an analytical tool for characterizing whole cell walls and isolated cell wall fractions [61,62] and has been used for quantification of features of lignin including S/G/H ratios and the relative abundance of monolignol side-chain inter-unit linkages [63,64] as well as identification of novel structures due to perturbations in lignin biosynthesis [60]. The aromatic regions of 2D ^13^ C-^1^ H HSQC spectra for hybrid and bm3 corn stover before and after AHP pretreatment are depicted in Figure 7. The ^13^ C-^1^ H peak assignments made based on previous studies [62] and by comparison with the literature [61,63,64] and signals are normalized to the S_2/6_ peak. Signal intensities of olefinic carbons originating from either *p*-hydroxycinnamates (*i.e. p*CA and FA) decrease with pretreatment, which is consistent with our Py-GC/MS data indicating *p*-hydroxycinnamate removal during pretreatment. While “semi-quantitative” at best, this data is used to provide a third estimation method for S/G ratio of the lignin remaining in the cell wall. These S/G ratios were computed as the integrated volume of the S_2/6_ peak, corrected by dividing by 2, and dividing by the volume of the G_2_ peak. The results are presented in Table 2 and compared with the other two methods used in this work. A key finding is that the NMR method determines higher S/G ratios than either of the other methods and that the apparent S/G ratio increases with pretreatment, especially for the hybrid corn stover.

**Table 2:** Comparison of Methods for Estimation of S/G Ratios in the Residual Cell Wall

	Thioacidolysis-GC/MS	Py-GC/MS	2D HSQC NMR
Hybrid corn stover, untreated	0.88 ± 0.097	0.64 ± 0.019	1.31
Hybrid corn stover, treated	1.01 ± 0.044	0.68 ± 0.031	2.42
Inbred bm3 stover untreated	0.26 ± 0.061	0.20 ± 0.015	0.24
Inbred bm3 stover, treated	0.27 ± 0.059	0.23 ± 0.036	0.27

In the aliphatic region of the spectrum (not shown), the peaks for 2-acetyl β-D xylopyranose units and 3-acetyl β-D-xylopyranose units were observed to disappear following pretreatment while the β-D-xylopyranose was retained, indicating that acetyl groups on xylans were cleaved by alkali and solubilized in both hybrid corn stover and bm3 stover which is consistent with HPLC data from our lab (not shown).

## Discussion

Lignocellulosic biomass represents a vast resource of reduced carbon that is overwhelming used for its existing structural value (as fiber and as a building material), for combustion, or as livestock forage rather than for the value of its chemical constituents. This is due to the challenges of deconstruction and fractionation of the biopolymer components of the plant cell wall, in particular due to the impact of lignin. Lignin structure, composition, and distribution within the cell wall of plants has obvious important economic implications for ruminant digestibility, chemical pulping, and cellulosic biofuels based on the biological conversion of plant cell wall polysaccharides. This work addresses this challenge by applying the generated data to relate glucan enzymatic digestibility to lignin and plant cell wall properties, to better characterize and understand the mechanism of AHP pretreatment for improved cell wall deconstruction, and to compare three analytical platforms (HSQC NMR, Py-GC/MS, and thioacidolysis) for the analysis of grass lignins.

### Relating cell wall enzymatic digestibility to lignin properties

A number of recent reviews have provided an insightful assessment for what properties comprise a promising lignin phenotype for ease of polysaccharide enzymatic deconstruction [10,24,31]. From these it is possible to distinguish four lignin properties that may impact the enzymatic digestibility and/or the pretreatability of a plant cell wall: (1) total lignin content; (2) ratio of monolignols and the corresponding degree of condensed linkages between monolignols; (3) the location of the lignin; and (4) for grasses, the abundance of *p-*hydroxycinnamates and their makeup. To illustrate the impact of these lignin properties in the context of our work, a number of studies can be highlighted. For dicots, where ferulate crosslinking is not as significant a feature of cell walls, strong negative correlations have been shown between initial lignin content and cellulolytic enzyme digestibility for dilute acid pretreated alfalfa (*Medicago sativa* L) [3] and hot water [4] or dilute acid [65] pretreated *Populus* spp. For grasses, however, Iiyama and Lam [66] found that *in vitro* ruminant digestibility of untreated rye grass (*Lolium* sp*.*) displaying a diverse range of initial lignin contents (18–25% w/w) was not dependent on initial lignin content while the initial content of ferulate bridges was strongly, negatively correlated to digestibility. Similar trends have been shown previously in the literature [67] where both the cellulase degradability and the *in vitro* ruminant digestibility of bm3 and a “normal” cultivar of maize were strongly negatively correlated to the total content of alkali-labile *p-*hydroxycinnamates independent of lignin content over a wide range of Klason lignin contents (5.5% to 14.2%). Zhang *et al.*[49] found that cellulolytic enzyme digestibility in 10 maize lines could be strongly negatively correlated to Klason lignin content (in the range of 12.4–15.2%), total etherified and esterified *p*-coumaric acid content, and β-O-4 abundance, but not to etherified ferulate content and S/G ratio. Other work has found unpretreated grasses with initial lignin contents in the range of 2–8% to be a strong predictor of *in vitro* ruminant digestibility [68]. Taken together these results may indicate that for grasses at higher Klason lignin contents, cell wall digestibility is a strong function of the content of *p-*hydroxycinnamates, while at lower lignin contents, Klason lignin seems to be a better predictor of digestibility. Lignin removal or redistribution has an obvious impact of polysaccharide accessibility and enzymatic digestibility. Acid chlorite delignification of orchardgrass (*Dactylis glomerata* L) was found to significantly improved its rate of *in vitro* rumen digestibility [69]. Yang and Wyman [6] showed that lignin removal in corn stover by hot water pretreatment in a flow-through reactor could be correlated to enzymatic digestibility, while hot water batch pretreatment could achieve comparable enzymatic digestibility without concomitant lignin removal. Because these pretreatments are performed above the glass transition temperature of lignin, the lignin is presumably relocalized outside the cell wall. Properties of lignin (*e.g.* β-O-4 content, free phenolics, S/G ratio, and ferulate content) other than those that are important for untreated cell wall digestibility may become important for pretreatment or delignification and result in improved digestibility after pretreatment.

Our results in Figures 1,2,3 and 4 indicate that the relationship between lignin content, ferulate and *p*-coumarate content (indirectly inferred from pyrolytic 4-vinylguaiacol and 4-vinylphenol abundance), and glucan enzymatic digestibility are all correlated. In particular it can be seen that functionalities between these properties are that Klason lignin and *p-*hydroxycinnamate content are linearly correlated which reveals their concurrent removal from the cell wall during AHP pretreatment. While it is not possible to determine directly which of these is the causal factor, it can be proposed that a combination of ferulate crosslink destruction and β-O-4 scission and a concomitant increase in free phenolics allows for lower molecular weight oligomers of lignin to be solubilized (but not necessarily significantly degraded) and removed from the cell wall. As stated previously, one interpretation of the digestibility “threshold” value in Figure 1 between a Klason lignin content of 10–15% may be the point where the internal environment of the cell wall becomes hydrophilic enough to allow water (and enzyme) penetration. Grabber *et al.*[16] identified a similar digestibility threshold value for enzymatic sugar release at approximately 15% lignin content in plant cell cultures artificially lignified with *p*-hydroxycinnamate esters.

The relationship between S/G ratio and enzymatic digestibility is less straightforward than the total lignin content due to multiple confounding factors including the fact that the largest differences in S/G ratios are between dicots which typically contain the highest S/G ratios have significantly different cell wall organization and structures and grasses or gymnosperms (softwoods) which typically contain a high fraction of guaiacyl units in their lignins. The S/G ratios in grasses and herbaceous dicots have been proposed to be correlated to the susceptibility of the plant cell wall to enzymatic deconstruction either positively, negatively, or not at all as reviewed by Méchin *et al.*[24]. As another example, after hot water pretreatment, cellulolytic glucose release was higher for natural *Populus trichocarpa* variants with higher S/G ratios [4] while total lignin content was a good predictor of cellulose conversion only at S/G ratios of less than 2.0. For alfalfa cell walls pretreated with dilute acid, large differences in enzymatic saccharification efficiencies were observed between various transgenic lines with significantly altered monolignol biosynthesis pathways, and in lines containing high H content, S/G ratio alone didn’t correlate with sugar yield [3]. For hot water-pretreated *Arabidopsis*, cell walls with higher S/G ratio gave a much higher glucose yield [70]. In grasses, Chen *et al.*, [14] correlated higher S/G ratios in untreated tall fescue (*Festuca arundinacea*) to decreased *in vitro* ruminant digestibility, although this result was confounded by total lignin content since increasing S/G was also correlated to increased total lignin content during plant maturation. Based on a study of 22 inbred maize lines, including four bm3 lines, Méchin [24] proposed that increasing the S/G ratio in grasses would improve the cellulolytic enzyme digestibility of grass cell walls and that *p*-coumarate acylation of syringyl moieties improved digestibility by decreasing interaction with carbohydrates. Our data in Figures 5,6 and 7 presenting the relative abundance of monolignols and their alteration and response to AHP pretreatment does not provide conclusive evidence of any strong influence of S/G ratio on lignin removal by pretreatment or on enzymatic conversion to glucose for any of the four grasses tested.

### Mechanisms of pretreatment by AHP against plant cell wall polymers

AHP pretreatment solubilizes a fraction of the lignin in the cell wall which, by itself, improves enzymatic glucan digestibility. Based on our data in Figure 6 we can propose that AHP pretreatment destroys alkyl-aryl ether linkages and potentially results in recondensation reactions. In comparing the impacts of other pretreatments on lignin, Glasser *et al.*[71] found similar results where steam explosion, dilute sulfuric acid, and organosolv pretreatments result in the destruction of alkyl-aryl ethers which lowered lignin monomer and dimer yields following permanganate oxidation and decreased the degree of polymerization of lignin remaining in the cell wall. They also found that considerable recondensation took place following dilute acid pretreatment. Our thioacidolysis lignin monomer yields for AHP pretreatment were significantly lower than the permanganate oxidation lignin monomer plus dimer yields of Glasser *et al.*[71] for other pretreatments (including only one grass, barley straw) and may indicate that AHP pretreatment is particularly effective at β-O-4 scission.

During an AHP pretreatment performed at a pH near the pK_a_ of H_2_O_2_ (pK_a_ = 11.5), hydrogen peroxide is decomposed to several important lignin-acting species including the perhydroxyl anion (OOH^–^) and by a secondary decomposition to hydroxyl (·OH) and superoxide anion (·O_2_^-^) radicals [36]. This β-O-4 scission by alkaline hydrogen peroxide is known from the literature on oxidative delignification and pulp bleaching [72]. In alkaline hydrogen peroxide bleaching, which is performed at a pH lower than 11.5, the perhydroxyl anion is presumably the dominant reactive oxidant rather than oxidative radical species. The perhydroxyl anion is proposed to attack conjugated carbonyl bonds and is important for pulp brightening during bleaching rather than delignification [86]. However, it has been shown that alkaline hydrogen peroxide bleaching can destroy internal alkyl-aryl ether bonds in lignin, while destruction of C-C bonds in the aliphatic region is possible in lignin subunits containing an adjacent phenolic hydroxyl moiety [72]. Because of this, alkaline hydrogen peroxide delignification is improved in lignins with a high phenolic hydroxyl content [73]. While initially grass lignins contain high phenolic hydroxyl contents, destruction of alkyl-aryl ethers in lignin would generate even more free phenolic groups and improve the solubilization of lignin in alkali and would presumably result in more sites of attack for delignification reactions.

It is also well-accepted that during alkaline oxygen or hydrogen peroxide bleaching, oxygen radical species are capable of lignin side chain cleavage and aromatic ring opening reactions [74,75] that would result in lignin depolymerization and increasing lignin hydrophilicity. At a pH close to 11.5 or at lower pH in the presence of Fenton’s-inducing transition metals, free radical chemistry becomes an important contribution to oxidative lignin reactions. Recent work has demonstrated selective scission of β-O-4 bonds in aspen lignin by brown rot fungi followed by a concomitant increase in benzaldehydes, benzoic acids, and phenylglycerols in the side chain regions of the monolignols resulting from these cleavages [76]. The proposed mechanism was through the Fenton’s generation of hydroxyl radicals from hydrogen peroxide which may be similar to the mechanism of AHP pretreatment.

In addition to alkyl-aryl ether scission, a wide range of reactions with lignin can take place during AHP pretreatment that increase lignin’s hydrophilicity [74]. Recently, alkaline hydrogen peroxide bleaching of eucalyptus Kraft lignin was found to carboxylate the α-C [54] of the lignin side chain region using NMR. In addition to the previously identified delignification, increasing lignin hydrophilicity (*e.g.* through carboxylation) is another proposed mechanism for enzymatic digestibility improvement by AHP, whereby the mechanism of improved digestibility could be through a combination of increasing cell wall water swelling with ensuing improvement in enzyme penetration into the cell wall and through a decrease enzyme adsorption onto less hydrophobic lignins [77]. Recently, lignin sulfonation or lignin removal and carboxylation through acid chlorite or AHP delignification were all found to improve the enzymatic digestibility of SO_2_-impregnated, steam-pretreated softwood presumably due to increased cell wall hydrophilicity [38] imparted by lignin functionalization. Nakagame *et al.*[7] utilized dehydrogenative polymer (DHP) lignins synthesized from ferulic acid and coniferyl alcohol to determine that the increasing carboxylic acid content of the artificial lignins reduced the non-productive binding of cellulases to improve cellulolytic activity.

### Comparison of lignin methods

Methods for determining the relative abundance of monomers in lignin include, among others, wet chemical lignin degradation techniques such as thioacidolysis [51], CuO oxidation [48], permanganate oxidation [71] and whole-cell wall methods such as analytical pyrolysis [78] and NMR [61-64]. One challenge to lignin characterization is that each method will have its own drawbacks and qualifications which, at a minimum, should be acknowledged when they are applied. As one example, degradative lignin analysis methods generally overestimate S-lignin [64] since these are less condensed and more prone to release monomers. As another example, *p*-hydroxycinnamates should ideally be distinguished from other monolignols in grasses when using NMR or analytical pyrolysis since response factors are significantly different [42]. Using GC/MS analysis of the products of CuO lignin degradation, the 3 pools of monolignols (H, G, S) as well as *p*-hydroxycinnamic acids were distinguished for white rot fungi degradation of wheat straw [47] or wheat straw cell wall fractions [48]. These were compared with results for analytical pyrolysis, which did not distinguish *p*-hydroxycinnamates from other lignins and found that the G + FA fraction was consistently significantly higher by analytical pyrolysis than by CuO degradation, presumably due to the disproportionate contribution by ferulates. This finding is in confirmation of our results in Figure 5 comparing S/G ratios where Py-GC/MS overestimates the guaiacyl fraction. One solution to this problem that has been proposed is to perform pyrolysis in the presence of tetramethylammonium hydroxide (TMAH) which yields distinctive markers for *p*-hydroxycinnamates [42] and allows estimation of their abundance relative to other lignin-derived aromatics. Determination of S/G ratios in dicots, where *p*-hydroxycinnamates are relatively insignificant, has resulted in much better agreement between methods. Recently, Py-GC/MS, thioacidolysis-GC/MS, and HSQC NMR have been used in parallel to characterize the lignins in woody [63,64] and herbaceous [79] dicots with good agreement between S/G ratios estimated by each method. Our results in Figure 4 and Table 2 highlights this difficulty in obtaining convergent results between methods for grasses where *p*-hydroxycinnamates confound the analysis, although the results are likely to be internally consistent.

## Conclusions

A number of notable findings were made in this work. The first is that total lignin content, pyrolytic 4-vinylguaiacol fractional yield (as a proxy for ferulate content), and pyrolytic 4-vinylphenol yield (as a proxy for *p*-coumarate content) were demonstrated to be negatively correlated to glucan digestibility for four grasses. Correlations were found between lignin content and glucan digestibility for these four species of grasses under different AHP pretreatments approximating a sigmoidal function with significant increases in digestibility occurring between 10 and 15% lignin content. We suspect that this threshold value may represent a critical level for cell wall hydrophobicity. The higher lignin content of the switchgrass was presumably the reason for its increased resistance to both pretreatment and digestibility. Another finding is that while S/G ratios for the grasses tested ranged from 0.2 to 2.2 as quantified by thioacidolysis, this parameter did not have any apparent quantifiable impact on either AHP pretreatment or enzymatic glucan digestibility although other significant differences in lignins between plant types may mask the effect of S/G ratio.

Another contribution of this work are the findings that the lignins of brown midrib corn stovers were significantly more condensed than a commercial hybrid corn stover (which is known from the literature) and that pretreatment with alkaline hydrogen peroxide under conditions that solubilizes approximately 50% of the lignin results in the fraction of condensed lignin increasing from 88–95% w/w to 99% w/w. This has the implication that, in addition to alkaline saponification of ester-linked *p*-hydroxycinnamates, one of the major impacts of AHP pretreatment is to decrease the β-O-4 content of the lignin remaining in the cell wall and potentially to induce lignin condensation reactions with the net result of dramatically decreasing the lignin monomers releasable by thioacidolysis.

Additionally, this work compared three methods for S/G ratio estimation in untreated and AHP-pretreated grasses for the first time and highlighted the limitations and challenges due to the confounding influence of *p*-hydroxycinnamates. Specifically, Py-GC/MS results indicated that the majority of pyrolyzable aromatics from grass lignins are likely *p*-hydroxycinnamates which are liberated disproportionately to their abundance rather than S, G and H lignin units. Removal of the 4-vinylguaiacol contribution still resulted in significantly lower S/G ratios by pyrolysis than by thioacidolysis or whole-cell wall NMR. However, the results do indicate that methods should be internally consistent and yield relatively similar trends between biomass samples and between pretreatment conditions.

## Methods

### Biomass

The biomass used in this study included switchgrass (*Panicum virgatum* cv. Cave-In-Rock) obtained from the Michigan Biotechnology Institute (Lansing, MI) and corn stovers (*Zea mays* L.) obtained from Natalia de Leon (University of Wisconsin, Madison). The corn stovers included a commercial hybrid (Pioneer Hi-Bred 36 H56) and two near-isogenic inbred lines of W64A maize containing the brown midrib alleles for bm1 and bm3 and were generated by introduction of the bm1 and bm3 mutations into the W64A background with 8 backcrosses followed by 6 self-pollinations. Biomass was initially milled with a Wiley MiniMill (Thomas Scientific) to pass a 2 mm screen and air-dried to ~5% moisture.

### AHP pretreatment, composition analysis, and enzymatic glucan digestibility

The biomass samples were subjected to pretreatments corresponding to “low NaOH” (0.1 g NaOH/g biomass), “high NaOH” (2.2 g NaOH/g biomass), and pH 11.5 (corresponding to slightly more than 0.1 g NaOH/g biomass) where the pH was adjusted by addition of 5 M NaOH during pretreatment. The H_2_O_2_ loading was 0.125 g H_2_O_2_/g biomass with additional conditions for hybrid corn stover using loadings of 0.25 and 0.50 g H_2_O_2_/g biomass at a controlled pH of 11.5. Biomass concentration was 2% (w/w) biomass in 100 mL total volume, and the pretreatment was performed for 24 hours in a temperature-controlled incubator at 30°C with orbital shaking at 170 rpm. Pretreatments were performed in duplicate and the untreated biomass was used as the control. Following pretreatment, biomass was subjected to vacuum filtration with simultaneously washing by deionized water until neutral, then was air-dried to ~5% moisture content. The pretreated biomass was subsequently ball-milled with a QIAGEN TissueLyser II equipped with 25 mL stainless steel jars and 20 mm diameter stainless steel balls at 25 Hz for 2 minutes with liquid nitrogen cooling. Compositional analysis was performed according to Sluiter *et al.*[80] with the difference that an Aminex HPX-87 H (Bio-Rad, Hercules, CA) column was used. Duplicate compositional analysis was performed for each pretreatment condition and the results are presented on a total composition basis rather than an extractives-free basis. Mass decrease as a result of AHP pretreatment was determined gravimetrically after washing and filtration of the residual insoluble material. For enzymatic digestibility determination, the washed and pretreated biomass samples were incubated at 50°C for 24 hours with Accellerase 1500 (Genencor Inc., Palo Alto, CA) at a loading of 50 mg protein/g glucan, which was chosen with the assumption that this would be above a “saturation” limit for enzyme loading which might give the highest possible conversions for a pretreatment condition such that only substrate effects could be evaluated. The conditions for hydrolysis were 10% solid loading at 5 mL total volume in 0.05 M Na-citrate buffer pH 4.8 for 24 hours. The enzymatic yield of glucose was determined by the HPLC analyzable glucose concentration after incubation divided by the original glucan content in the pretreated samples. Our previous work has demonstrated that approximately 5% of the glucan content may be solubilized during AHP pretreatment [81] possibly as sucrose, β-glucans, starch, or by oxidative modification of glucan.

### Thioacidolysis

Thioacidolysis was used to estimate the relative abundance and total yield of β-O-4 linked S, G, and H monolignols according to Foster *et al.*[82]. A 60 mg aliquot of the dried biomass was first ball milled in the iWall grinding and feeding robot using three 5 mm stainless steel ball bearings. Using the ball-milled biomass, three replicate assays of 2 mg each were weighed into glass screw cap reaction vials. To the vials, 200 μL of anhydrous 1,4-dioxane containing 2.5% (v/v) borontrifluoride diethyl etherate and 10% (v/v) ethanethiol was added and incubated at 100°C for 4 hours in a heating block. After cooling the samples on ice, 150 μL of 0.4 M sodium bicarbonate was added and then the reaction is extracted with 1 mL of water and 0.5 mL of ethyl acetate. A 150 μL aliquot of the organic phase was transferred to a 2 mL vial and evaporated under a gentle stream of dry air. An addition of 200 μL of dry acetone was added and evaporated twice to help rid the samples of residual water. The dried monomer residue was dissolved in 500 μL of ethyl acetate and transferred to a GC vial to which 20 μL of pyridine and 100 μL of N,O-bis(trimethylsilyl)acetamide is added. The reaction was left to incubate at room temperature for 2 hours before injecting onto the GC/MS (Agilent 6890 GC/Agilent 5975B MSD, Column: Agilent HP-5MS). To determine the quantitative yield of the monomers, synthetic thiol derivatives of the S, G, and H lignin monomers were graciously provided by Dr. John Ralph (University of Wisconsin, Madison) and an alternate method (manuscript in preparation) was adapted from Foster et al. [82] and Robinson and Mansfield [51] for quantitative analysis. Mass yields of thioacidolysis-liberated monomers were determined by conversion from the molar concentrations of the standards using the following conversion factors for the assumption that the monolignol is involved in only β-O-4 linkages: syringyl, 223 g/mol; guaiacyl, 192 g/mol; *p*-hydroxyphenyl, 161 g/mol. All samples were run in triplicate.

### Analytical pyrolysis

Ball-milled control and pretreated samples (500–1000 μg) were pyrolyzed in a quartz tube in a CDS AS 5250 Pyroprobe (Chemical Data Systems Analytical, Inc., Oxford, PA) at 650°C for 20 seconds at a filament heating rate of 1000°C/s using helium as the carrier gas with a flow rate of 1 mL/min. The sample was carried onto a 60 m × 0.25 μm × 0.25 μm Restek 1701 column fitted in a Shimadzu GC/MS-QP5050 with a 100 split ratio. The temperature was programmed to rise from 40°C to a final temperature of 270°C at 8°C/min, and held at that temperature for a total run time of 45 minutes. The injector and detector temperatures were set at 280°C. Mass spectra were recorded in electron ionization mode for m/z 40 to 400. Peaks were quantified by integrated area of each peak divided by the total peak area and the average of duplicates were taken, and identified based on the compound library of Shimadzu GCMSsolution software and by comparison to the NIST database or the literature [39] for 4-vinylphenol. The majority of the pyrolytic compounds in those chromatograms were identified with compound similarities greater than 80%.

### Cell wall 2D HSQC NMR profiling

The pre-milled commercial hybrid and bm3 corn stover samples were sequentially extracted by 70% ethanol, methanol/chloroform (1:1 v/v) and acetone. Approximately 30–50 mg of extractives-free sample were ball-milled for a total of 15 min in a Retsch Mixer Mill MM-200 (Retsch GmbH, Düsseldorf, Germany) and dissolved in anhydrous perdeuterated pyridinium chloride/DMSO-d_6_ bisolvent system (700 mg, 1:3 w/w) for 2D heteronuclear single quantum correlation (HSQC) ^13^ C-^1^ H NMR experiments which were carried out at 65 °C in a Bruker Avance 400 MHz NMR spectrometer equipped with a z-gradient triple-resonance probe [62,83]. A ^1^*J*_CH_ coupling constant of 145 Hz was used for all the HSQC experiments. The HSQC acquisitions were carried out with the following conditions: 10-ppm spectra width in F2 (^1^ H) dimension with 2048 data points, 210-ppm spectra width in F1 (^13^ C) dimension with 128 data points, 1.5-s pulse delay, and 380 scans. The DMSO solvent peak (*i.e.* δ_C_ 39.5 ppm and δ_H_ 2.5 ppm) was used for the chemical shift calibration. Bruker TopSpin 2.1 software was used to process NMR spectra data.

## Abbreviations

AHP, Alkaline hydrogen peroxide; FA, Ferulic acid; G, Guaiacyl; GC/MS, Gas chromatography mass spectrometry; H, p-hydroxyphenyl; HPLC, High performance liquid chromatography; HSQC, Heteronuclear single-quantum correlation; MW, Molecular weight; NMR, Nuclear magnetic resonance; pCA, p-coumaric acid; Py, Pyrolysis; S, Syringyl.

## Competing interests

The authors declare that they have no competing interests.

## Authors’ contributions

DBH conceived the work, DBH and ML wrote the manuscript, ML performed the pretreatment, composition analysis and enzymatic digestibility analysis, SK and ML performed the analytical pyrolysis, *CF* and ML performed the thioacidolysis analysis, YP performed the HSQC NMR analysis, and all authors reviewed this manuscript and provided input and corrections.
